# Optimized Transgene Delivery Using Third‐Generation Lentiviruses

**DOI:** 10.1002/cpmb.125

**Published:** 2020-09-28

**Authors:** Katherine P. Gill, Mark Denham

**Affiliations:** ^1^ Danish Research Institute of Translational Neuroscience (DANDRITE), Nordic EMBL Partnership for Molecular Medicine Aarhus University Aarhus Denmark; ^2^ Department of Biomedicine Aarhus University Aarhus Denmark

**Keywords:** high titer, lentiviral production, lipofection, ultracentrifugation, third‐generation

## Abstract

The lentivirus system enables efficient genetic modification of both dividing and non‐dividing cells and therefore is a useful tool for elucidating developmental processes and disease pathogenesis. The development of third‐generation lentiviruses has resulted in improved biosafety, low immunogenicity, and substantial packaging capabilities. However, because third‐generation lentiviruses require successful co‐transfection with four plasmids, this typically means that lower titers are attained. This is problematic, as it is often desirable to produce purified lentiviruses with high titers (>1 × 10^8^ TU/ml), especially for in vivo applications. The manufacturing process for lentiviruses involves several critical experimental factors that can influence titer, purity, and transduction efficiency. Here, we describe a straightforward, stepwise protocol for the reproducible manufacture of high‐titer third‐generation lentiviruses (1 × 10^8^ to 1 × 10^9^ TU/ml). This optimized protocol enhances transgene expression by use of Lipofectamine transfection and optimized serum replacement medium, a single ultracentrifugation step, use of a sucrose cushion, and addition of a histone deacetylation inhibitor. Furthermore, we provide alternate methods for titration analyses, including functional and genomic integration analyses, using common laboratory techniques such as FACS as well as genomic DNA extraction and qPCR. These optimized methods will be beneficial for investigating developmental processes and disease pathogenesis in vitro and in vivo. © 2020 The Authors.

**Basic Protocol 1**: Lentivirus production

**Support Protocol**: Lentivirus concentration

**Basic Protocol 2**: Lentivirus titration

**Alternate Protocol 1**: Determination of viral titration by FACS analysis

**Alternate Protocol 2**: Determination of viral titration by genome integration analysis

## INTRODUCTION

Third‐generation lentiviruses are desirable for gene modification because they are capable of transducing a broad range of cell types that are difficult to transfect by traditional methods (Fraley, Subramani, Berg, & Papahadjopoulos, 1980; Graham & Van der EB, 1973; Kawai & Nishizawa, 1984; Komatsu et al., 1992; McCutchan & Pagano, 1968; Neumann, Schaefer‐Ridder, Wang, & Hofschneider, 1982; Pagano & Vaheri, 1965), can stably integrate into the genome and provide long‐term transgene expression, and are safer than previous generations. Production of high‐titer (>10^8^ TU/ml) third‐generation lentiviruses depends on the efficient simultaneous delivery of four plasmids into a packaging cell, low cytotoxicity, and the ability to successfully concentrate the virus without mechanical shearing. Lentivirus titer is determined by several critical experimental variables, including packaging cell type and confluency, transfection method, serum concentration, size of the transfer vector, total amount of DNA delivered, and relative proportions of envelope, packaging, and transfer vectors. The human embryonic kidney cell line HEK293T/17 is widely used as the packaging cell line because of its high lentiviral productivity (Tomás, Rodrigues, Alves, & Coroadinha, 2013). HEK293T/17 cells harbor SV40 large T antigen, which recognizes and binds to the SV40 origin to increase plasmid DNA amplification and virus output (Gama‐Norton et al., 2011).

Traditional transfection methods for lentivirus production use calcium phosphate (CaPi) or polyethylenimine (PEI). A drawback of these common methods is the requirement for large quantities of packaging cells to produce high‐titer viruses. For preclinical studies and gene therapies, it is important to know the concentration of vector particles transduced into a target cell. Furthermore, it is desirable to increase the titer and transduction efficacy without increasing the volume of lentivirus solution administered to a tissue or animal.

**Figure 1:**
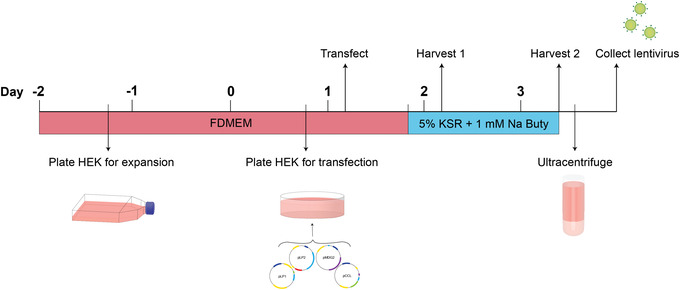
Experimental timeline for lentiviral production. HEK293T/17 cells are plated in the afternoon of day ‐2 for expansion. Approximately 48 hr later, the HEK cells are trypsinized and seeded into 10‐cm^2^ dishes for transfection. In the morning of day 1, the HEK cells are transfected with Opti‐MEM containing the Lipofectamine and DNA complex. Transfection media are removed 6 hr later and replaced with 5% KSR containing 1 mM sodium butyrate (denoted by the abbreviation “Na Buty”). The first lentivirus harvest is performed in the morning of day 2, 24 hr post‐transfection. The second lentivirus harvest is performed around midday of day 3, 52 hr post‐transfection. After ultracentrifuge concentration, the purified lentiviral particles can be aliquoted and stored.

Here, we report an optimized stepwise protocol for the reproducible manufacture of high‐titer (1 × 10^8^ to 1 × 10^9^ TU/ml) third‐generation lentiviruses (Basic Protocol 1). The modified protocol uses Lipofectamine 3000 transfection for optimal transfection of HEK293T/17 cells, combined with an optimized serum replacement medium to avoid the complement system, a single concentration step and a sucrose cushion to reduce viral shearing, and addition of a histone deacetylation inhibitor (sodium butyrate) for stable gene expression. Lentivirus production takes 3 days in total (Fig. 1). To titer the lentivirus, we describe three possible methods: (i) functional titration of lentiviruses containing a reporter by manual calculation (see Basic Protocol 2), (ii) FACS analysis (see Alternate Protocol 1), or (iii) genome integration titration analysis using qPCR (see Alternate Protocol 2). Further, we describe an additional concentration method (see Support Protocol) to boost the lentivirus titer if required. An added advantage of the straightforward stepwise procedures described in this article is the use of common laboratory techniques and equipment to produce and titer lentiviruses. Public access to improved lentiviral manufacturing procedures, such as the methods reported here, will be crucial for investigating developmental processes and disease pathogenesis in vitro and in vivo.

Highlights of the approach are as follows:

- A single ultracentrifugation step with high‐titer lentivirus yield.
- Use of KSR medium in place of serum‐containing medium to avoid the complement system.
- Use of a sucrose cushion and omission of a vortexing step to minimize mechanical stress.

*NOTE*: All steps are performed in a biosafety level 2 (BSL‐2) laboratory following standard regulatory procedures.

*NOTE*: All solutions and equipment coming into contact with cells must be sterile, and proper sterile technique should be used accordingly.

*NOTE*: All culture incubations are performed in a humidified 37°C, 5% CO_2_ incubator unless otherwise specified.

## Basic Protocol 1 LENTIVIRUS PRODUCTION

This protocol describes the reagents and steps to generate a single batch of high‐titer third‐generation lentivirus based on ultracentrifuge rotor volume capacity. Prior to lentivirus production, the HEK293T/17 cells must be expanded to ensure a sufficient quantity of cells for transfection on day 1 (Fig. 1). For this, HEK cells are seeded into three T175 flasks on day ‐2 and expanded for 3 days. Transfection is performed in the morning of day 1 using the Lipofectamine 3000 kit (Thermo Fisher Scientific), followed by a medium change 6 hr later. Two lentivirus harvests are conducted, on day 2 (24 hr post‐transfection) and day 3 (52 hr post‐transfection), to coincide with maximal gene expression and lentivirus production by HEK cells (Fig. 1). Ultracentrifugation of lentivirus supernatant is performed on day 3 to collect purified lentivirus that can be stored until titration analysis.

### Materials

HEK293T/17 cells (ATCC, cat. no. CRL‐11268)
Dulbecco's phosphate‐buffered saline (PBS; without calcium and magnesium; Gibco, cat. no. 14190094), room temperature and 4°C
0.05% (w/v) trypsin‐EDTA (Gibco, cat. no. 25300054)
FDMEM medium (see recipe)
KSR medium (see recipe)
0.5 M sodium butyrate (Sigma‐Aldrich, cat. no. B5887)
Sucrose cushion solution (see recipe), 4°C
Dry ice

15‐ and 50‐ml conical tubes
Standard tabletop centrifuge, room temperature and 4°C
Hemocytometer
T175 flasks (Nunc, cat. no. 10246131)
10‐cm^2^ tissue culture–treated dishes (BD, cat. no. 10212951)
0.45‐μm Stericup filters (Sarstedt, cat. no. 83.3941)
UC tubes: 38.5‐ml, 25 × 89–mm, thin‐walled, open‐top polypropylene ultracentrifuge tubes (Beckman Coulter, cat. no. 326823), 4°C
10‐ml plastic pipets
AH‐629 swinging‐bucket rotor (six 36‐ml buckets; Thermo Fisher Scientific, cat. no. 54284) or as specified for ultracentrifuge, 4°C
Sorvall™ WX+ Ultracentrifuge (Thermo Fisher Scientific, cat. no. 75000100) or equivalent, 4°C
Kimwipes (KIMTECH) or equivalent
1.5‐ml Eppendorf tubes (e.g., VWR, cat. no. 211‐2116)

Additional reagents and equipment for preparing transfection media A and B (see recipes)

*NOTE*: Filtered or non‐filtered pipet tips should be used where appropriate.

### Day ‐2: HEK293T/17 culture and expansion

1 Passage HEK293T/17 cells by aspirating culture medium and washing with 3 ml PBS.
  Maintain HEK293T/17 cells by culturing them in FDMEM medium in a T175 flask until ∼80% confluency. Depending on the cell quality and proliferation rate, the cells will need approximately 3 to 4 days before the next passage.
2 Aspirate PBS and add 2 ml of 0.05% trypsin‐EDTA to flask. Incubate for 3 to 5 min at room temperature.
3 Mechanically dislodge cells by tapping the flask and then add 8 ml FDMEM medium to neutralize trypsin.
4 Transfer HEK cell suspension to a 15‐ml conical tube. Centrifuge cell suspension for 3 min at 300 × *g*, room temperature. Remove supernatant carefully from the cell pellet and then resuspend in 5 ml FDMEM.
5 Take an aliquot of cell suspension and perform a cell count with a hemocytometer.
6 Seed 5 × 10^6^ HEK cells in a total of 25 ml FDMEM per T175 flask. Incubate HEK cells in a humidified incubator at 37°C, 5% CO_2_, for 2 days.
  Approximately three T175 flasks are required.
  HEK expansion can be performed over a 48‐ to 72‐hr period, depending on the proliferation rate. Note that 82.5 × 10^6^ cells are required for step 7.

### Day 0: HEK293T/17 plating for transfection

7 Trypsinize expanded HEK cells by aspirating the medium and washing with 3 ml PBS. Aspirate PBS and then add 2 ml of 0.05% trypsin‐EDTA to flask. Incubate for 3 to 5 min at room temperature.
8 Mechanically dislodge cells by tapping the flask and then neutralize trypsin by adding 8 ml FDMEM.
9 Transfer cell suspension to a 15‐ml conical tube. Centrifuge cell suspension for 3 min at 300 × *g*, room temperature. Carefully remove supernatant from the cell pellet and then resuspend in 5 ml FDMEM.
10 Take an aliquot of the HEK cell suspension and perform a cell count with a hemocytometer.
11 Plate HEK cells at a density of 7.5 × 10^6^ cells in a total of 10 ml FDMEM per 10‐cm^2^ tissue culture–treated dish for ∼16 hr.
  Prepare 11 dishes in total.
  Plate HEK cells ∼16 hr prior to transfection (steps 12 to 19) to ensure 80% to 90% confluency at the time of transfection.

### Day 1: HEK293T/17 transfection

12 Prepare transfection medium A (see recipe).
13 Prepare transfection medium B (see recipe).
14 Combine transfection media A and B and incubate at room temperature for 10 min.
15 Remove 4 ml FDMEM from each 10‐cm^2^ dish.
16 Carefully pipet 3 ml of combined transfection media A and B onto each dish.
17 Incubate for 6 hr at 37°C, 5% CO_2_.
18 Remove transfection media.
19 Add 10 ml KSR medium supplemented with 20 μl of 0.5 M sodium butyrate (1 mM final) to each dish and incubate for an additional 18 hr at 37°C, 5% CO_2_.

### Day 2: Harvest 1 (24 hr post‐transfection)

20 Twenty‐four hours post‐transfection, Collect all lentiviral supernatant from dishes and transfer into 50‐ml conical tubes.
21 Centrifuge 10 min at 2000 × *g*, 4°C.
22 While waiting, slowly add 10 ml KSR medium (room temperature) supplemented with 20 μl of 0.5 M sodium butyrate (1 mM final) to the HEK dishes. Return HEK cells to the incubator.
23 After centrifugation, transfer lentivirus supernatant into new 50‐ml conical tubes. Store overnight at 4°C.

### Day 3: Harvest 2 (52 hr post‐transfection)

24 Fifty‐two hours post‐transfection, Collect all lentiviral supernatant from dishes and transfer into 50‐ml conical tubes.
25 Centrifuge 10 min at 2000 × *g*, 4°C.
26 Combine all lentivirus supernatants (from Harvest 1/step 23 and Harvest 2/step 25) into a 500‐ml 0.45‐µm Stericup filter. Filter lentivirus supernatant with a vacuum pump or pipettor.
  The total volume should be ∼220 ml.
27 Place filtered supernatant in the Stericup on ice.

### Ultracentrifugation

**Figure 2:**
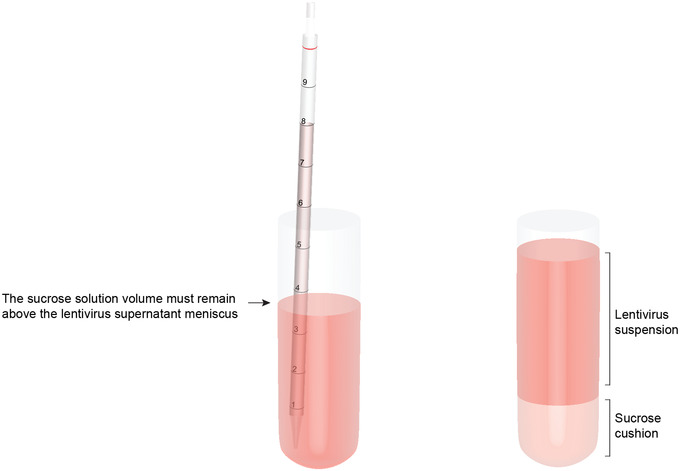
Diagram illustrating the technique to create a sucrose cushion in the ultracentrifuge tubes. Using a long plastic pipet, slowly add 2 ml sucrose solution to the base of the tube, maintaining a volume in the pipet higher than the meniscus of the lentiviral supernatant.

28 Transfer 30 ml filtered lentivirus supernatant into each pre‐chilled UC tube (six tubes total).
29 Slowly add 2 ml cold sucrose cushion solution directly to base of three UC tubes using a 10‐ml plastic pipet filled containing 8 ml cold sucrose cushion solution (see Fig. 2). After filling the first three UC tubes, use a new 10‐ml plastic pipet to repeat this for the remaining three tubes.
  To produce a dense, discontinuous sucrose cushion without bubbles, the viscous solution must be pipetted slowly and with a volume in the pipet that remains above the KSR medium to prevent backflow of the lentivirus supernatant into the pipet. See Figure 2.

30 Transfer UC tubes into the pre‐chilled rotor buckets (AH‐629 swinging‐bucket rotor or as specified for the ultracentrifuge).
31 Fill UC tubes with any remaining supernatant (see step 27), KSR medium, or PBS to ensure that the tube volume is ∼36 ml.
32 Tightly screw lids onto the rotor buckets and place into pre‐chilled rotor.
33 Release vacuum on the pre‐chilled ultracentrifuge (Sorvall™ WX+ Ultracentrifuge or equivalent). Carefully lower rotor into the ultracentrifuge. Wipe any condensation from rotor chamber.
34 Ultracentrifuge lentiviral supernatants for 90 min at 91,000 × *g*, 4°C.
35 Remove rotor and buckets. Immediately pour contents of each UC tube into a waste container and then place each tube upside‐down on clean paper towels.
  A small pellet containing virus particles may be visible at the base of each tube.
36 Quickly remove excess supernatant from the UC tube walls using clean Kimwipes or equivalent. Repeat.
37 Add 80 μl cold PBS to each UC tube. Place UC tubes on ice.
  Lentivirus particles should remain chilled henceforth.
38 Working quickly with one UC tube at a time, carefully resuspend virus pellet in the PBS using a pipet.
  Viral pellets may be difficult to see. To ensure resuspension, repeatedly pipet the PBS up and down along the inner surface of the tube base in an attempt to wash adhered viral particles off the surface. Avoid creating bubbles, as these may shear the virus particles.
39 Transfer lentivirus supernatant from each UC tube into a single 1.5‐ml Eppendorf tube and place on ice.
  The total lentivirus particle volume should be ∼600 μl, and the expected titer will be in the range of 1 × 10^8^ to 1 × 10^9^. To further concentrate the lentivirus, a second ultracentrifugation can be performed (see Support Protocol). The expected titer after a second ultracentrifugation will be in the range of 1 × 10^9^ to 1 × 10^10^.
40 Aliquot small volumes of lentivirus supernatant into separate labeled 1.5‐ml Eppendorf tubes and place immediately on dry ice. Store lentivirus aliquots ≤1 year at −80°C.
  Lentivirus stocks are recommended to be used within 1 year because longer storage may reduce transduction efficiency. It is recommended to aliquot the lentivirus into small volumes (e.g., 10 μl) to avoid repeated freeze‐thaw of lentivirus, which can also reduce transduction efficiency.

## LENTIVIRUS CONCENTRATION

A second ultracentrifugation step may be performed with the purified lentivirus from step 39 of Basic Protocol 1 to further concentrate the lentiviral particles. This may be necessary if the initial titer is predicted to be below expectations due to large transfer plasmid size or where an extremely high titer and low viral volumes are optimal, such as for in vivo applications.

### Additional Materials (also see Basic Protocol 1)

Lentivirus particle supernatant (see Basic Protocol 1, step 39)

UC tubes: 13.5‐ml, 16 × 76–mm, thin‐walled, open‐top polypropylene ultracentrifuge tubes (Beckman Coulter, cat. no. 326814)
SW 41 Ti swinging‐bucket rotor (six 13.2‐ml buckets; Beckman Coulter, cat. no. 331362) or as specified for ultracentrifuge, 4°C

1 Place lentivirus particle supernatant from step 39 of Basic Protocol 1 into a UC tube containing 6 ml KSR medium.
2 Carefully add 0.5 ml sucrose cushion solution to base of the tube using a 2‐ml pipet tip.
3 Prepare a second UC tube with equal weights of KSR medium and PBS as a balance.
4 Transfer the two UC tubes into two rotor buckets.
5 Top up UC tubes to ∼12.8 ml with KSR medium or PBS. Screw lid tightly onto each bucket.
6 Ensure that all six buckets are hooked into pre‐chilled rotor (SW 41 Ti swinging‐bucket rotor or as specified for the ultracentrifuge), with the two filled buckets placed opposite to each other.
7 Perform steps 34 to 40 from Basic Protocol 1.

## Basic Protocol 2 LENTIVIRUS TITRATION

This protocol describes the steps to titer the lentivirus manufactured in Basic Protocol 1 or the Support Protocol. The method involves a serial dilution of the lentivirus stock and transduction of HEK293T/17 cells to generate a standard curve of concentrations. A total of 16 to 18 hr prior to transduction, the HEK cells are seeded onto six‐well tissue culture plates at a relatively low density to maximize transduction efficiency. The following morning, separate wells of HEK cells are transduced with different concentrations of lentivirus from the serial dilution. The transduced HEK cells are incubated for 48 hr to ensure gene expression, and then the concentration is analyzed.

The concentration (transduction efficiency) of the lentivirus is determined using a functional titration method. Here, for functional assessment, the lentivirus of interest needs to contain a fluorescent reporter (e.g., GFP) that is used to calculate the relative proportion of transduced cells by manual counting. This method requires use of a fluorescent microscope equipped with a camera. Imaging analysis software, such as the freeware ImageJ (see Internet Resources), is recommended to assist with manual counting and to minimize human error. Alternatively, FACS analysis (Alternate Protocol 1) can be performed using a lentivirus containing a fluorescent reporter (e.g., GFP), or qPCR analysis can be conducted to assess the relative lentivirus copy number compared to a control lentivirus of known concentration (Alternate Protocol 2).

### Materials

HEK293T/17 cells (ATCC, cat. no. CRL‐11268)
FDMEM medium (see recipe)
Lentivirus particle supernatant (see Basic Protocol 1 or Support Protocol)
PBS (without calcium and magnesium; Gibco, cat. no. 14190094)

6‐well tissue culture–treated plates, treated (Thermo Fisher Scientific, cat. no. 140675)
Upright fluorescent microscope equipped with camera

**Figure 3:**
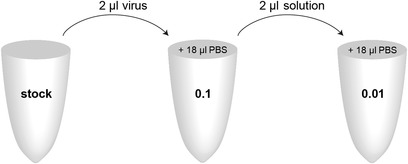
Serial dilution series of lentivirus stock. Prepare a 0.1 dilution by transferring 2 μl of the undiluted virus stock into a single 1.5‐ml Eppendorf tube containing 18 μl PBS. Mix well. Using a new pipet tip, transfer 2 μl of this dilution into a new 1.5‐ml Eppendorf tube containing 18 μl PBS to prepare the 0.01 dilution.

1 Seed HEK293T/17 cells onto 6‐well tissue culture–treated plates at a density of 0.5 × 10^6^ cells/well in 2 ml FDMEM medium approximately 16 to 18 hr prior to transduction.
2 Thaw an aliquot of lentivirus particle supernatant on ice and prepare 0.1 and 0.01 dilutions of virus in PBS (see Fig. 3).

**Figure 4:**
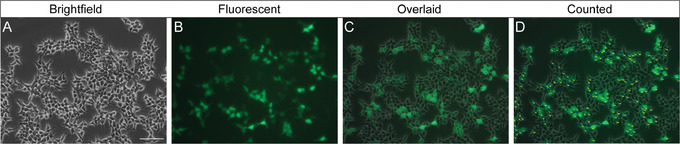
Representative brightfield images (**A**) and fluorescent images (**B**) of HEK293T/17 cells transduced with a dilution of lentivirus with a GFP reporter. An overlaid image (**C**) can be generated to assist with identifying cell boundaries in the fluorescent channel. The labeling and measure functions in ImageJ are used to count cells (**D**). Scale bar: 100 µm.

3 Add 1 μl undiluted virus to a single well of 6‐well plate from step 1.
4 Add 1 μl of each serial dilution (0.1 and 0.01) to two separate wells and 3 μl of each dilution (0.1 and 0.01) to an additional two separate wells. Keep remaining well with no virus as a negative control (0 μl).
  The result will be five wells containing 1 μl of 0.3, 0.1, 0.03, or 0.01 dilution of the virus stock.
5 Incubate for 48 hr at 37°C, 5% CO_2_.
  Those interested in using FACS or qPCR for the titration analysis, instead of the manual counting approach described here (see steps 6 to 8), would now continue with Alternate Protocol 1 or 2, respectively.
6 Capture a minimum of four randomized images per well with an upright fluorescent microscope equipped with a camera using appropriate excitation/emission filters at 20× magnification (emission of ∼512‐µm width).
  Images should be representative of the transduction efficiency in each condition. Record both brightfield and fluorescent images.
7 Perform total and fluorescent cell counts using brightfield and fluorescent images, respectively (see Fig. 4).
  Use of image‐processing software such as ImageJ will make it easier to record cell counts. Generate an overlaid brightfield/fluorescent image to help identify cell boundaries and facilitate accurate fluorescent cell counts. To do so, first open the image in ImageJ. Use the zoom function if the cells are small or their boundaries are difficult to determine. Select the “Multi‐point” tool from the panel and manually select each individual cell. Once the entire image has been counted, go to “Analyze” > “Measure” to generate a report of the results, which can then be exported as a text delimited file.

**Figure 5:**
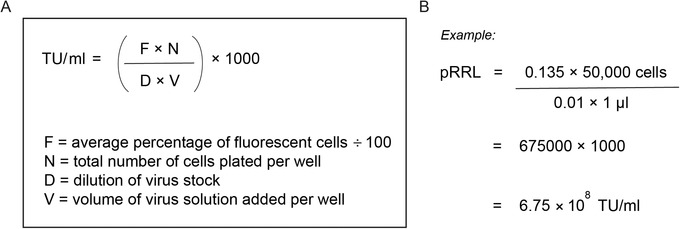
Formula for determining lentiviral transducing units per milliliter (TU/ml) (**A**). An example of the pRRL‐PGK‐EGFP plasmid titration (**B**).

8 Calculate functional transducing units (TU/ml) as described in Figure 5.
  The TU/ml should be calculated from wells with a 10% to 15% transduction rate to ensure that a single lentiviral particle is transducing a single cell. Wells with transduction rates below or above this range should not be used to calculate the TU/ml, as this will not be an accurate estimate of transduction efficiency.

## Alternate Protocol 1 DETERMINATION OF VIRAL TITRATION BY FACS ANALYSIS

An alternate method for functional titration of the lentivirus manufactured in Basic Protocol 1 is to use FACS analysis. This method, just as the one described in Basic Protocol 2, requires that the lentivirus contains a fluorescent reporter to assess the number of functional viral particles based on the proportion of fluorescent cells. As a FACS machine will isolate and measure all cells expressing the GFP reporter, strict gating will need to be performed during analysis to exclude cell doublets that may contain a non‐transduced cell. Gating is determined using the positive‐ and negative‐control samples included in the lentivirus serial dilution from Basic Protocol 2.

### Additional Materials (also see Basic Protocol 2)

0.05% (w/v) trypsin‐EDTA (Gibco, cat. no. 25300054)
8% (w/v) paraformaldehyde (PFA; in ddH_2_O; Sigma‐Aldrich, cat. no. 158127), 4°C

1.5‐ml Eppendorf tubes (e.g., VWR, cat. no. 211‐2116)
Standard tabletop centrifuge
Vortex
Flat‐bottom 96‐well tissue culture–treated plate (polystyrene; Corning, cat. no. 3598) or equivalent
Flow cytometer (e.g., Quanteon)

**Figure 6:**
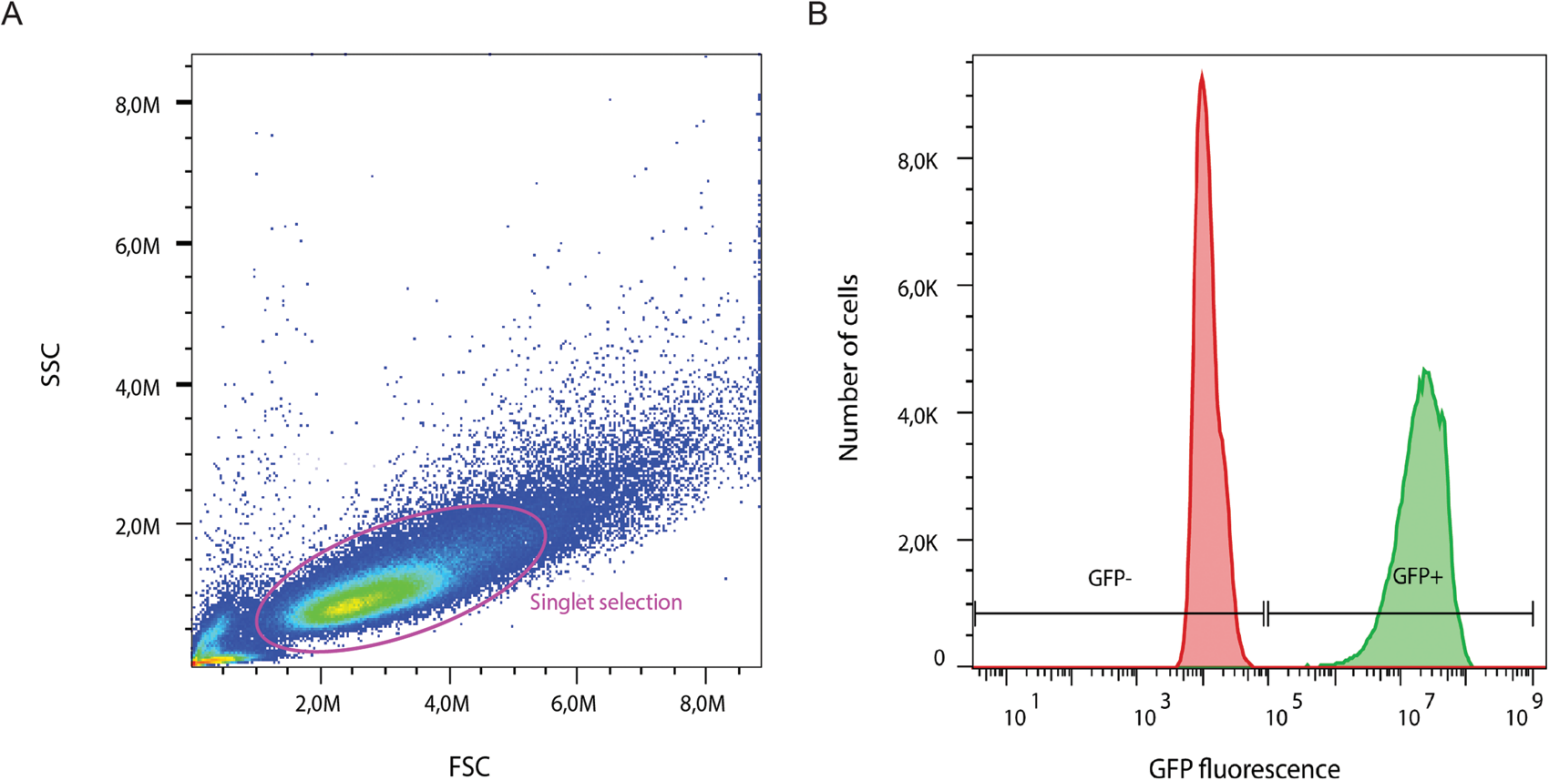
Representative flow cytometry analysis of a GFP reporter lentivirus. Singlet cells were isolated from the entire population by strict gating, in pink (**A**). The positive‐control (green) and negative‐control (red) samples were overlaid and used to set appropriate gates for GFP positivity (+) or negativity (–) (**B**). SSC, side scatter; FSC, forward scatter. GFP fluorescence intensity is shown in arbitrary units.

1 Follow steps 1 to 5 from Basic Protocol 2. Trypsinize transduced cells by aspirating the medium from each well, washing once with 0.5 ml PBS, and then adding 0.5 ml of 0.05% trypsin‐EDTA for 3 min at room temperature. Mechanically dislodge cells by tapping the plate, neutralize trypsin with 0.5 ml FDMEM medium, and transfer cell suspension from each well into a separate labeled 1.5‐ml Eppendorf tube.
2 Centrifuge tubes for 3 min at 300 × *g*, room temperature.
3 Remove supernatant, resuspend cell pellet with 0.5 ml PBS, and centrifuge 3 min at 300 × *g*, room temperature. Repeat.
4 Remove supernatant and add 200 μl cold PBS to cell pellet. Using a pipet, carefully mix cell suspension. Add 200 μl cold 8% PFA and incubate on ice for 10 min.
5 Add 400 μl cold PBS, centrifuge 3 min at 300 × *g*, room temperature, and then remove supernatant. Repeat.
6 Add 200 μl cold PBS to each tube. Keep chilled at 4°C until FACS analysis.
7 Vortex tubes to ensure a thoroughly mixed single‐cell suspension.
8 Transfer each sample to a single well of a flat‐bottom 96‐well tissue culture–treated plate or equivalent.
9 Load plate into a flow cytometer and follow manufacturer's guidelines for analysis.
  Ensure that the single‐cell population is isolated by adjusting the forward‐ and side‐scatter settings. Gate the cell population strictly with the positive‐control (1 μl) and negative‐control (0 μl) samples (Fig. 6).

10 Export flow cytometer analysis results. Calculate transduction efficiency using the equation shown in Figure 5.

## Alternate Protocol 2 DETERMINATION OF VIRAL TITRATION BY GENOME INTEGRATION ANALYSIS

An alternative method for titering the lentivirus (compared to Basic Protocol 2 and Alternate Protocol 1) is to calculate, by qPCR, the relative number of genomic integration events in a cell line that has been transduced with a range of viral volumes. This method requires that a control lentivirus of known titer is used as a comparison. To perform qPCR analysis, genomic DNA (gDNA) is extracted from the transduced HEK cells (see Basic Protocol 2, step 5). qPCR analysis is performed with the gDNA samples and with primers targeting the lentivirus sequence (LV2) and an internal control gene (albumin). Using the lentivirus of known titer, a concentration curve is created and used to calculate the relative number of DNA sequences of LV2 in the lentivirus of unknown titer.

### Additional Materials (also see Basic Protocol 2 and Alternate Protocol 1)

Proteinase K (New England Biolabs, cat. no. P8107S)
Genomic DNA lysis buffer (see recipe)
Double‐distilled water (ddH_2_O)
qPCR reagents:
  GoTaq qPCR Master Mix (2×; Promega, cat. no. A6002)
  Primers targeting LV2 and albumin gene (100 μM; synthesized by Sigma‐Aldrich; see Table 1)
  Nuclease‐free water (Ambion, cat. no. AM9930)
  **Table 1:** qPCR Primers and Working Concentrations

Target gene*a*	Sequence	Concentration (nM)
LV2 FWD	5′‐ACTTGAAAGCGAAAGGGAAAC‐3′	50
LV2 REV	5′‐CACCCATCTCTCTCCTTCTAGCC‐3′	50
Albumin FWD	5′‐TTTGCAGATGTCAGTGAAAGAGA‐3′	300
Albumin REV	5′‐TGGGGAGGCTATAGAAAATAAGG ‐3′	300

a FWD, forward primer; REV, reverse primer.

55°C and 100°C water baths
96‐well reaction plate (MicroAmp^TM^ Fast Optical 96‐Well Reaction Plate, Thermo Fisher Scientific, cat. no. 4346907)
qPCR machine (e.g., 7500 Fast Real‐Time PCR System, Thermo Fisher Scientific)

*NOTE*: Experiments involving PCR require extremely careful technique to prevent contamination.

### gDNA extraction

1 Follow steps 1 to 5 from Basic Protocol 2. Trypsinize transduced cells by aspirating the medium from each well and washing once with 0.5 ml PBS.
2 Remove PBS, add 200 μl of 0.05% trypsin‐EDTA, and incubate for 3 min at room temperature. Then, mechanically dislodge cells by tapping the plate.
3 Neutralize with 800 μl FDMEM medium and transfer cell suspension from each well into a separate labeled 1.5‐ml Eppendorf tube.
4 Centrifuge tubes for 6 min at 300 × *g*, room temperature.
5 Remove supernatant and resuspend cell pellet with 0.5 ml PBS. Centrifuge 3 min at 300 × *g*, room temperature.
6 Remove 90% of supernatant (∼980 μl) and carefully mix remaining volume with a pipet to resuspend the cell pellet.
7 Prepare a master mix containing 2 μl proteinase K and 18 μl genomic DNA lysis buffer per sample. Add 20 µl master mix to each cell suspension and incubate in a 55°C water bath for 30 min.
8 Remove tubes from the water bath and add 180 μl ddH_2_O to each tube. Incubate for 10 min in a 100°C water bath.
9 Chill gDNA samples on ice for 5 min and then store at 4°C until qPCR analysis.
  Samples can be stored long term at −20°C.

### qPCR analysis

10 Place all qPCR reagents on ice.
11 Prepare a PCR master mix consisting of 1× GoTaq qPCR Master Mix, primers (Table 1), and nuclease‐free water up to 9 μl total for each reaction.
  Prepare one master mix per primer pair. Here, one mix is needed for the albumin primers and one for the LV2 primers.
12 Pipet 1 μl gDNA (from step 9) per well into a 96‐well reaction plate.
  Samples are tested in triplicate per gene. Use the same tip for one sample; change tips between samples.
13 Pipet 9 μl albumin or LV2 master mix (see step 11) into appropriate wells to a final volume of 10 μl.
  Change tips between wells.
14 Pipet 9 μl master mix per gene (see step 11) into triplicate wells with 1 μl nuclease‐free water as a negative control.
15 Centrifuge plate for 3 min at 1000 × *g*, room temperature.
16 Program qPCR machine with for the following amplification:

**Table:** 

Initial step:	10 min	95°C (activation)
40 cycles:	15 s	95°C (denaturation)
	1 min	60°C (annealing/extension)
Final step:	*x*	*Tm* (melting‐curve stage)
  x = time.
  Tm = melting temperature.
17 Export raw data and calculate titer of the new lentivirus relative to the reference lentivirus, as shown in Figure 7.

**Figure 7:**
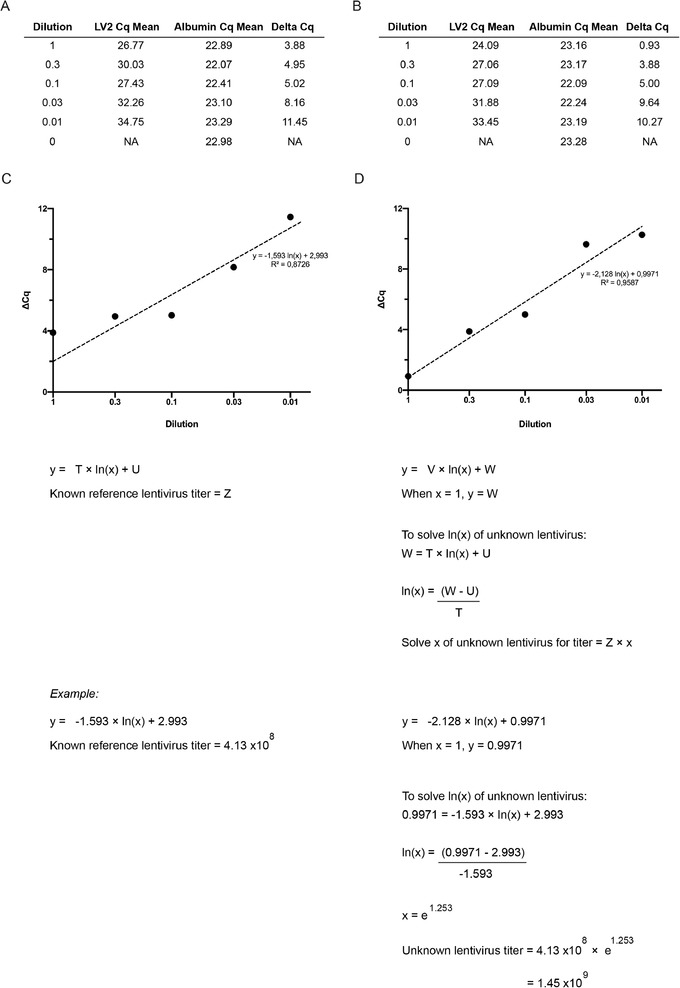
qPCR analysis for lentivirus titration. Representative triplicate Cq values for the LV2 and albumin genes are averaged to calculate the Cq mean for a serial dilution of lentivirus of known (**A**) or unknown (**B**) titration. A delta (Δ) Cq is calculated by subtracting the LV2 Cq mean from the albumin Cq mean. The Cq mean values for the lentivirus of known (**C**) and unknown (**D**) titration are plotted on an XY graph against dilution value. A natural log regression analysis is performed, and the unknown variables are used to calculate the unknown lentivirus titer. The letters (T‐W, Z) denote variables to be solved using the formula.

## REAGENTS AND SOLUTIONS

### FDMEM medium

432.5 ml Dulbecco's modified Eagle's medium (DMEM; Gibco, cat. no. 11960044)
5 ml GlutaMAX (Gibco, cat. no. 35050038) (1% final)
5 ml 100× MEM non‐essential amino acids (NEAA; Gibco, cat. no. 11140035) (1× final)
5 ml 100× sodium pyruvate (Gibco, cat. no. 11360039) (10 mM final)
2.5 ml penicillin‐streptomycin (pen‐strep; 10,000 IU/ml penicillin, 10,000 μg/ml streptomycin; Gibco, cat. no. 15140122) (50 U/ml final)
Mix all reagents listed above and filter with 0.22‐µm Stericup filter (Sarstedt, cat. no. 83.3941)
Add 50 ml fetal bovine serum (FBS; ultra‐low endotoxin; Biowest, cat. no. ALB‐S1860‐500) (10% final)
Store ≤1 month at 4°C
  This medium recipe is sufficient for a single batch of lentivirus. Pre‐warm the medium to room temperature prior to application to cells.

### Genomic DNA lysis buffer

5 mM EDTA
50 mM Tris⋅HCl, pH 8.2
100 mM NaCl
0.5% (w/v) SDS
Distilled H_2_O
Store ≤1 year at room temperature

### KSR medium

460 ml DMEM (Gibco, cat. no. 11960044)
5 ml GlutaMAX (Gibco, cat. no. 35050038) (1× final)
5 ml 100× NEAA (Gibco, cat. no. 11140035) (1× final)
5 ml 100× sodium pyruvate (Gibco, cat. no. 11360039) (10 mM final)
Mix all reagents listed above and filter with 0.22‐µm Stericup filter (Sarstedt, cat. no. 83.3941)
Add 25 ml KnockOut Serum Replacement (KSR; Gibco, cat. no. 10828020) (5% final)
Store ≤1 month at 4°C
  This medium recipe is sufficient for a single batch of lentivirus. Pre‐warm the medium to room temperature prior to application to cells, unless stated otherwise.

### Sucrose cushion solution

50 mM Tris⋅HCl, pH 7.4
100 mM NaCl
0.5 mM EDTA
20% (w/v) sucrose
Store ≤1 year at room temperature

### Transfection medium A

16.5 ml Opti‐MEM reduced‐serum medium (Gibco, cat. no. 31985070), room temperature
451 μl Lipofectamine 3000 Reagent (from Lipofectamine 3000 kit, Thermo Fisher Scientific, cat. no. L3000075)
Prepare fresh immediately before use
  The Lipofectamine 3000 Reagent diluted in Opti‐MEM should be used within 15 min of preparation to avoid reduced transfection efficiency.
  This medium recipe is sufficient for a single batch of lentivirus. Pre‐warm the medium to room temperature prior to application to cells, unless stated otherwise.

### Transfection medium B

16.5 ml Opti‐MEM reduced‐serum medium (Gibco, cat. no. 31985070), room temperature
36.3 µg pMD2.G plasmid (Addgene, cat. no. 12259)
36.3 µg pRSV‐Rev plasmid (Addgene, cat. no. 12253)
72.6 µg pMDLg/pRRE plasmid (Addgene, cat. no. 12251)
47.3 µg pRRL‐PGK‐EGFP‐WPRE plasmid (Addgene, cat. no. 12252) or equivalent third‐generation lentivirus plasmid with gene of interest
385 μl P3000 Enhancer Reagent (from Lipofectamine 3000 kit, Thermo Fisher Scientific, cat. no. L3000075)
Prepare fresh immediately before use
  The order of addition is important. The plasmids should be added to the Opti‐MEM for dilution. The P3000 Enhancer Reagent should be added last.
  This medium recipe is sufficient for a single batch of lentivirus. Pre‐warm the medium to room temperature prior to application to cells, unless stated otherwise.

## COMMENTARY

### Background Information

Lentiviruses are widely used to deliver genetic material into host cells for stable transgene expression. Lentiviral vectors capitalize on the membrane fusion capabilities of recombinant lentiviruses, such as human immunodeficiency virus type 1 (HIV‐1), containing long terminal repeats and integrase, to utilize the host cellular machinery and stably integrate their genetic cargo into the host genome. Pseudotyping HIV‐1 with the G glycoprotein of vesicular stomatitis virus (VSV‐G) is commonly used to improve the tropism of typical retroviral vectors (Burns, Friedmann, Driever, Burrascano, & Yee, 1993; Yee et al., 1994) and has several added advantages. The VSV‐G envelope protein allows fusion with the cell membrane to facilitate viral entry into the host cell by endocytosis (Mastromarino, Conti, Goldoni, Hauttecoeur, & Orsi, 1987). The VSV‐G protein does not shed from the lentiviral particles and can endure shearing forces during ultracentrifugation, allowing concentration of the lentiviral particles (Burns et al., 1993). Further, VSV‐G can withstand several freeze‐thaw cycles (Verhoeyen & Cosset, 2004) and is known to improve infectivity (Maréchal, Clavel, Heard, & Schwartz, 1998).

Second‐generation lentiviruses were developed to reduce cytotoxicity and improve the biosafety of the original wild‐type HIV‐based vectors. In the second‐generation vector system, genes for all unnecessary accessory proteins (*vif*, *vpr*, *vpu*, and *nef*) were removed, and the HIV‐1 envelope was replaced with VSV‐G (Zufferey, Nagy, Mandel, Naldini, & Trono, 1997). Second‐generation lentiviruses require the delivery of a packaging plasmid containing *gag*, *pol*, *rev*, and *tat*; an envelope plasmid containing the *env* gene and a transfer plasmid containing a 5′LTR and 3′LTR; a *psi* packaging element; and the genetic material of interest. *Tat* transcribes the transfer plasmid for packaging from the 5′LTR. The biosafety of this system has been further improved in the third generation of lentiviruses by dividing the packaging plasmid into two (one encoding *gag* and *pol* and one encoding *rev*), removing *tat*, and altering the 3′ LTR, which limits self‐activation and re‐packaging of integrated genes (Zufferey et al., 1997; Naldini et al., 1996; Dull et al., 1998).

The lentivirus manufacturing protocol described in this article (Basic Protocol 1) utilizes the third‐generation vector system. Here, four plasmids are delivered: a packaging plasmid (pMDLg/pRRE) containing *gag* and *pol*, a reverse response element, and the integrase gene; a second packaging plasmid (pRSV‐Rev) containing an RSV promoter and the *rev* gene; the pMD2.G envelope plasmid, encoding the VSV‐G protein; and a fourth plasmid discussed below. The reverse response elements are an additional biosafety precaution, as they require *rev* to be expressed during virus production. The fourth plasmid, a transfer plasmid containing a fluorescent reporter under a constitutive promoter, provides a convenient tool to titrate the virus by visualizing the expression of the reporter and can be used in high‐throughput screening assays and in live‐cell analyses of gene modification. Two additional elements were incorporated into the transfer plasmid to improve the efficiency of transgene expression: the central polypurine tract (cPPT) upstream of the promoter and the woodchuck hepatitis virus (WHV) post‐transcriptional response element (WPRE) (Werner, Kraunus, Baum, & Brocker, 2004).

Traditionally, lentiviruses were manufactured using the CaPi and PEI methods. The CaPi method requires the precipitation of DNA using a solution of sodium phosphate, calcium chloride, saline, and glycerol (Graham & Van der EB, 1973). Although this technique is cost effective, its efficiency is sensitive to small changes in pH. The more recent PEI method uses a polymeric transfection reagent with high protonability and wide tropism (Boussif et al., 1995). With sufficient PEI, the DNA will condense and form a PEI/DNA complex that is transported by endosomes. Although the PEI reagent can withstand a wide pH range, the major disadvantage of this technique is the requirement for large quantities of plasmid DNA. Overall, both the CaPi and the PEI methods have limitations for efficient lentivirus production because they require large quantities of packaging cells to generate lentiviruses of high titer.

Liposome‐mediated transfection (lipofection) methods require liposomes to form a cationic lipid complex with plasmid DNA. The Lipofectamine reagents (Thermo Fisher Scientific) are based on this technology (Felgner et al., 1987). The Lipofectamine 3000 kit (Thermo Fisher Scientific), used here (Basic Protocol 1), provides superior transfection with plasmid DNA and an increase in lentiviral supernatant yield and titer (Shi et al., 2018). Our lentivirus production protocol (Basic Protocol 1) was further optimized by replacing FDMEM, containing serum, with KSR medium to avoid the mammalian complement system. This is based on well‐known reports that serum activates the complement system to degrade retroviruses (Cooper, Jensen, Welsh, & Oldstone, 1976; Welsh, Cooper, Jensen, & Oldstone, 1975). This simple modification overcomes the need for further pseudotyping with an envelope plasmid containing a complement regulatory protein, such as delay‐accelerating factor (Guibinga & Friedmann, 2010). Further, we implemented use of a sucrose cushion during ultracentrifugation to improve virus recovery following concentration (al Yacoub, Romanowska, Haritonova, & Foerster, 2007). Ultracentrifugation exerts shearing forces on the envelope protein (Burns et al., 1993) and internal viral core (Kim & Lim, 2017) that can reduce lentivirus recovery. Indeed, other mechanical stress factors such as vortexing and bubble formation, which we have minimized in Basic Protocol 1, can reduce viral stability.

Lentiviruses are a preferred vector choice for the efficient and stable delivery of a transgene in vitro and in vivo, with a wide range of applications. Due to their broad tropism, lentiviruses can be used to deliver genetic material into a variety of cell types with high efficiency, including post‐mitotic neurons (Naldini et al., 1996; Zufferey et al., 1997). Because of their long‐term and stable integration capability, lentiviruses can be used to generate stable cell lines with an antibiotic selection cassette (LaGory et al., 2015) or reporter (Knudsen et al., 2018), providing an indispensable tool to elucidate gene function in development and disease. Lentiviruses can be packaged with short‐hairpin RNA (for knock‐down experiments), the open reading frame of a gene of interest, and the CRISPR‐Cas9 system with multiple guide RNAs (Yiu, Tieu, Nguyen, Wong, & Smit‐McBride, 2016) for genome editing. Further, lentiviruses can be designed to include ubiquitous or tissue‐specific promoters for the generation of transgenic animals (Lois, Hong, Pease, Brown, & Baltimore, 2002) and induced pluripotent stem cell lines (Habekost, Jørgensen, Qvist, & Denham, 2019; Sommer et al., 2009). The therapeutic potential of lentiviruses has also been exploited, including vaccine (Sanders et al., 2016) and oncolytic (Peng et al., 2002) strategies. Several preclinical and clinical studies are already underway. The long‐term safety and efficacy of using integrating lentiviruses as a method of patient gene therapy remain uncertain. However, early results from a number of clinical trials to treat Parkinson's disease (Palfi et al., 2014), human ß‐thalassemia (Negre et al., 2016), and cystic fibrosis (Marquez Loza, Yuen, & McCray, 2019) are promising. Thus, protocols for large‐scale manufacture of purified, high‐titer lentiviruses are sought after for both preclinical and clinical applications. The stepwise protocol described in this article (Basic Protocol 1) can achieve reproducible production of high‐quality, high‐titer lentiviruses using common techniques and equipment.

### Critical Parameters

#### HEK293T/17 maintenance

For optimum lentivirus manufacture (Basic Protocol 1), HEK293T/17 cells must be maintained consistently, following recommendations by ATCC (see Internet Resources). Due to a rapid proliferation rate, HEK cells should be passaged at a minimum of once per week, reaching a maximum of 80% confluency. ATCC recommends no more than 10 passages over a 2‐month period for a HEK cell culture. Medium should be renewed every 3 to 4 days. Poor HEK cell quality can limit transfection efficiency and lentivirus titer yield.

#### Lentivirus production

Sodium butyrate must be added to the KSR medium during HEK cell transfection (Basic Protocol 1). Addition of histone deacetylase (HDAC) inhibitors, such as sodium butyrate, is known to enhance stable gene expression during lentivirus manufacture (Karolewski, Watson, Parente, & Wolfe, 2003; Sena‐Esteves, Tebbets, Steffens, Crombleholme, & Flake, 2004). HDACs inhibit transcription by catalyzing the deacetylation of histones, thereby condensing chromatin structure (Butler & Bates, 2006; Chen & Townes, 2000; Chen, Zhao, & Zhao, 2015). Inhibition of HDACs facilitates reopening of the chromatin and increases transcription (Butler & Bates, 2006).

#### Ultracentrifugation

Prior to beginning the second harvest (Basic Protocol 1), the ultracentrifuge should be pre‐chilled to 4°C. After loading the rotor bucket into the chilled ultracentrifuge, wipe the internal surfaces of the centrifuge to prevent condensation, as it will reduce the performance of the vacuum pump.

#### Lentivirus supernatant

After centrifuging the virus suspension, the UC tubes should be quickly removed from the rotor buckets, the medium discarded, and the tubes placed upside‐down onto sterile laboratory paper in a biohazard hood (Basic Protocol 1 and Support Protocol). This step is crucial, as extended time in the rotor may disturb the viral pellet, causing it to resuspend in the solution, thereby reducing the lentiviral titer. Work quickly and use clean Kimwipe tissues or equivalent to remove any liquid that drips down the shaft of the tube. While resuspending the pellet, take care not to create bubbles to minimize shearing forces, which can reduce lentivirus recovery.

#### FACS titration

The FACS results (Alternate Protocol 1) can be incorrectly analyzed if cell debris or doublets are quantified. Cell debris will have very small SSC and FSC values, whereas doublets will have very large values. Cell debris is a result of degradation and can be avoided by fixing freshly trypsinized cells. To ensure a single‐cell suspension, first vortex the cell pellet to prevent cell‐cell adhesion during fixation. Then, add the 8% PFA to fix the single‐cell suspension. Prior to flow cytometry analysis, resuspend the fixed, washed cells in a small volume (∼200 μl). During analysis, apply strict gating to ensure that only transduced singlets are measured, based on the fluorescent reporter. Singlets will have roughly proportionate SSC and FSC values.

#### qPCR analysis

Prior to qPCR analysis (Alternate Protocol 2), it is recommended to validate and optimize the LV2 and albumin primers. Primer sequences can first be checked using free primer design software, such as the OligoAnalyzer Tool (Integrated DNA Technologies; see Internet Resources), which contains algorithms to predict the likelihood of secondary structure formation, including primer dimers. Further, primers can be validated by gel electrophoresis or melting‐curve analysis via qPCR to detect primer dimers. Primer concentration can be optimized by qPCR to reduce the incidence of dimerization by generating a concentration standard curve against a fixed annealing temperature.

A melting‐curve analysis should be performed when analyzing the lentivirus titration samples to assess primer specificity. The PCR amplification curve is used to determine the cycle quantification value (Cq) for each sample. The Cq values of the LV2 gene are then normalized to the internal control gene albumin.

### Troubleshooting

Please see Table 2 for a list of common issues and potential solutions.

**Table 2:** Troubleshooting Guide for Lentivirus Production, Concentration, and Titration

Issue	Possible cause	Solution
Large amount of cell debris during LV*a* production	High cytotoxicity of plasmids	The HEK cell density may be too low. Check confluency prior to transfection. The amount of transfer plasmid containing the DNA of interest transfected may need to be optimized. Filter the LV supernatant with a 0.45‐μm Stericup filter.
Lower‐than‐expected LV titer	Poor quality of HEK cells (e.g., high passage number) Incorrect density of HEK cells before transfection (too low/high) Poor transfection efficiency	Thaw a lower passage of HEK cells or order a new batch from ATCC. Ensure an accurate cell count and “guestimate” the confluency prior to transfection. Thaw a lower passage of HEK cells or order a new batch from ATCC. The transfer plasmid size may be large. Consider delivery of two separate plasmids or repeat the ultracentrifugation step to concentrate (see Support Protocol). Ensure that sodium butyrate is added to the KSR medium.
	LV particle degradation	Minimize bubble formation and mechanical shearing during the LV supernatant collection step.
Low proportion of GFP‐positive cells	Low LV transduction efficiency	Ensure that the LV supernatant is kept cold during collection. Minimize repeated freeze‐thaw cycles. The HEK cell density may be incorrect. If the cells are too confluent, the LV particles will not transduce properly.
Difficulty manually counting transduced cells because cell boundaries are not clear	High HEK cell proliferation rate, with too many cells by the time of analysis	Reduce the HEK cell density before transduction. Create an overlaid image with both brightfield and fluorescent images to better see the cell boundaries while counting GFP‐positive cells. While using ImageJ, use the zoom tool to magnify the image.
Inconsistent albumin Cq values across sample sets	Improperly lysed gDNA samples	Ensure that the transduced cell pellet is mixed thoroughly before adding the lysis buffer; otherwise, the cells may remain attached in a clump and will not lyse, and gDNA will not be extracted. This will affect the gDNA concentration and the relative number of DNA sequences detected by qPCR.
Low LV2 Cq values	Improperly lysed gDNA samples	See above.
	Low LV transduction efficiency	See above.

a LV, lentivirus.

### Understanding Results

Using the optimized protocol with a single ultracentrifugation step (Basic Protocol 1), lentiviral titers in the range of 10^8^ to 10^9^ TU/ml are expected. Plasmids containing different lentiviral elements may yield differential results. For example, large transfer plasmid size is known to limit packaging efficiency and is sensitive to ultracentrifugation during viral particle concentration (al Yacoub et al., 2007).

### Time Considerations

As outlined in Figure 1, lentivirus production (Basic Protocol 1) takes 5 days in total, including 2 days of HEK cell expansion prior to the start of the protocol. A second ultracentrifugation (Support Protocol) may be performed, which takes an additional 3 hr. Functional titration of lentiviruses containing a reporter requires that HEK cells are cultured for 2 days after transduction, followed by approximately 2 to 4 hr of analysis, depending on whether manual calculation (Basic Protocol 2) or FACS analysis (Alternate Protocol 1) is performed. Genomic integration titration (Alternate Protocol 2) is an alternative technique following HEK cell transduction from Basic Protocol 2 that requires approximately 3 to 4 hr of analysis. A detailed breakdown of each protocol is shown below:

Basic Protocol 1:

- HEK plating for transfection: 0.5 hr.
- Lipofectamine reagent preparation: 1 hr (0.25 hr for incubation)
- Transfection: 6 hr.
- Medium changes (two): 0.5 hr.
- Harvest 1: 1 hr.
- Harvest 2 and ultracentrifugation preparation: 1.5 hr.
- Ultracentrifugation: 1.5 hr.
- Lentivirus preparation: 1.5 hr.

Support Protocol:

- Ultracentrifugation: 1.5 hr.
- Lentivirus preparation: 1.5 hr.

Basic Protocol 2:

- Transduction: 48.5 hr (0.5 hr preparation).
- Image analysis: 2 to 3 hr.

Alternate Protocol 1:

- FACS analysis: 1.5 hr (0.66 hr preparation).

Alternate Protocol 2:

- gDNA extraction: 1.5 hr.
- qPCR analysis: 2 hr (0.5 hr preparation).

### Author Contributions

**Katherine P. Gill**: Data curation; formal analysis; investigation; validation; writing‐original draft. **Mark Denham**: Conceptualization; funding acquisition; methodology; project administration; supervision; writing‐review & editing.
